# Point accuracy and reliability of an interstitial continuous glucose-monitoring device in critically ill patients: a prospective study

**DOI:** 10.1186/s13054-015-0757-4

**Published:** 2015-02-05

**Authors:** Roosmarijn TM van Hooijdonk, Jan Hendrik Leopold, Tineke Winters, Jan M Binnekade, Nicole P Juffermans, Janneke Horn, Johan C Fischer, Edmée C van Dongen-Lases, Marcus J Schultz

**Affiliations:** Department of Intensive Care, Academic Medical Center, University of Amsterdam, Meibergdreef 9, 1105 AZ Amsterdam, The Netherlands; Laboratory of Experimental Intensive Care and Anesthesiology (L · E · I · C · A), Academic Medical Center, University of Amsterdam, Meibergdreef 9, 1105 AZ Amsterdam, The Netherlands; Department of Clinical Chemistry, Academic Medical Center, University of Amsterdam, Meibergdreef 9, 1105 AZ Amsterdam, The Netherlands

## Abstract

**Introduction:**

There is a need for continuous glucose monitoring in critically ill patients. The objective of this trial was to determine the point accuracy and reliability of a device designed for continuous monitoring of interstitial glucose levels in intensive care unit patients.

**Methods:**

We evaluated point accuracy by comparing device readings with glucose measurements in arterial blood by using blood gas analyzers. Analytical and clinical accuracy was expressed in Bland-Altman plots, glucose prediction errors, and Clarke error grids. We used a linear mixed model to determine which factors affect the point accuracy. In addition, we determined the reliability, including duration of device start-up and calibration, skips in data acquisition, and premature disconnections of sensors.

**Results:**

We included 50 patients in whom we used 105 sensors. Five patients from whom we could not collect the predefined minimum number of four consecutive comparative blood draws were excluded from the point accuracy analysis. Therefore, we had 929 comparative samples from 100 sensors in 45 patients (11 (7 to 28) samples per patient) during 4,639 hours (46 (27 to 134) hours per patient and 46 (21 to 69) hours per sensor) for the accuracy analysis. Point accuracy did not meet the International Organization for Standardization (ISO) 14971 standard for insulin dosing accuracy but did improve with increasing numbers of calibrations and was better in patients who did not have a history of diabetes. Out of 105 sensors, 60 were removed prematurely for a variety of reasons. The device start-up time was 49 (43 to 58) minutes. The number of skips in data acquisition was low, resulting in availability of real-time data during 95% (89% to 98%) of the connection time per sensor.

**Conclusions:**

The point accuracy of a device designed for continuous real-time monitoring of interstitial glucose levels was relatively low in critically ill patients. The device had few downtimes, but one third of the sensors were removed prematurely because of unresolved sensor- or device-related problems.

**Trial registration:**

Netherlands Trial Registry number: NTR3827. Registered 30 January 2013.

**Electronic supplementary material:**

The online version of this article (doi:10.1186/s13054-015-0757-4) contains supplementary material, which is available to authorized users.

## Introduction

Handheld blood glucose meters or department-based blood gas analyzers are currently the preferred methods to measure blood glucose levels in intensive care unit (ICU) patients [1,2]. These intermittent glucose-monitoring techniques have variable accuracies [3] but foremost lack useful trending because of the interval between consecutive measurements. Continuous glucose monitoring (CGM) is suggested to increase practicalities and safety of insulin titration in ICU patients [1,4], in particular when targeting normal or near-normal blood glucose levels when hypoglycemic episodes can be expected [5-13].

Glucose oxidase technique-based interstitial CGM devices have been used before in diabetic patients outside the ICU setting [14]. It is uncertain, however, whether interstitial CGM devices are point accurate in critically ill patients [1]. An altered relationship between blood and interstitial fluid glucose levels during critical illness could affect the point accuracy of interstitial CGM to reflect the blood glucose level [15]. Several interstitial CGM sensor systems originally designed for non-ICU patients have been tested in the ICU setting in recent years [16-28]. Medtronic MiniMed (Medtronic Inc., Northridge, CA, USA) developed the Sentrino Continuous Glucose Management System, an interstitial CGM device that was especially designed for use in critically ill patients. This device was improved from previous models by creating the processor cable and pole-mounted monitor and by four sensing elements designed to increase responsiveness to glucose changes and to limit the influence from drug interactions.

The aim of this study was to test its point accuracy and reliability in a mixed medical-surgical ICU. We hypothesized that the device would provide an accurate reflection of the blood glucose level in ICU patients treated according to a local guideline for blood glucose control targeting blood glucose levels between 90 and 144 mg/dL. In addition, we determined its reliability, including duration of the device start-up, the need for calibration, skips in data acquisition, and number of and reasons for premature disconnections.

## Methods

### Study design and informed consent

This was an investigator-initiated observational trial. The Institutional Review Board of the Academic Medical Center (Amsterdam, The Netherlands) approved the study protocol (study ID: NL41498.018.12). Medtronic MiniMed provided three devices for the duration of the trial and the necessary sensors but had no influence on study design or study reporting. Patients or next of kin had to provide written informed consent before the start of any study-related procedure.

### Study population

Patients were recruited between October 2012 and February 2014 in a 30-bed mixed medical-surgical ICU of a large university hospital (Academic Medical Center). Patients were eligible for inclusion if they were at least 18 years old and had an anticipated life expectancy of more than 96 hours. Patients were excluded from participation if they had a platelet count of less than 30 × 10^9^/L, had participated in a trial testing an investigational product or treatment within the past 30 days, were pregnant, or had a suspected or diagnosed medical condition which in the opinion of the investigators prevented the patient from completing the study.

### Glucose control

ICU nurses performed glucose control with insulin by following a local guideline for blood glucose control targeting a blood glucose level between 90 and 144 mg/dL [29]. Insulin titration adjustments were based on sliding scales. The local guideline for blood glucose control dictated nurses to perform blood glucose measurements at least every 4 hours and more frequently if blood glucose levels were out of range or were expected to change rapidly. For details, see Additional file 1.

During the study, ICU nurses were not allowed to change insulin infusion rate based on the readings by the investigational device. However, they were allowed to perform additional blood glucose measurements if the device suggested rapid changes in the glucose level or when there was a trend toward hypoglycemia.

### The investigational device

The disposable glucose sensors of the device were glucose oxidase-based; each sensor had two probes, and each probe had two sensing elements. The individual measurement results were combined and displayed on the device monitor every minute. The signal was transmitted through the processor cable to the monitor. It was a single-patient single-use sensor, which could be used for up to 72 hours. The processor cable was reusable.

The sensor was inserted into the subcutaneous tissue by using two parallel introducer needles. The two needles automatically retracted when the introducer hub was pulled away from the sensor base; the sensor probes remained in the subcutis. Each new sensor needed calibration by using blood glucose levels after insertion and initialization and after 1 hour and 2 hours; thereafter, repeated calibrations were performed every 8 hours.

### Study procedures

Sensors were inserted into the subcutis of the thigh. Successive sensors could be used for 72 hours, depending on length of stay in the ICU, but never for longer than 30 days. Arterial blood glucose levels were measured by using RapidLab 1265 blood gas analyzers (Siemens Healthcare Diagnostics, The Hague, The Netherlands), which were used for calibrations of the device. Not only did ICU nurses provide the mandatory calibration blood glucose levels, but also the routinely obtained blood glucose levels (that is, blood glucose measurements which were not requested by the device for calibrations but were taken by the nurses as dictated by the local guideline for blood glucose control) were entered into the device as well. Therefore, these measurements were also used for calibrations of the device. If the device displayed a message requesting an additional non-routine calibration to resolve a sensor performance issue (that is, a ‘Poor Sensor Signal’ alert), the nurses were permitted to disregard manufacturer recommendation and remove sensors rather than enter the requested calibration.

Each day, the place of insertion was photographed and inspected for redness, bruises, and swelling. In case the patient was awake, we questioned the patient whether it was painful. Every item could be scored as ‘none’, ‘minor’, or ‘major’.

### Power calculation

We intended to enroll 50 patients to assess accuracy of the CGM device. With 50 patients, we expected to have at least 40,000 subcutaneous CGM device results and at least 1,200 blood glucose level measurements with the RapidLab 1265. Considering previous studies testing point accuracy, we assumed we would have a sufficiently high number of paired samples to enable evaluation of the point accuracy of the device.

### Analysis plan

The glucose data collected with each new sensor were downloaded from the device after use in a patient; the arterial blood glucose levels were downloaded from the patient data management system. The arterial blood glucose levels in the patient data management system were compared with the entries for calibrations into the device. In case of an entry error, defined as a difference between the arterial blood glucose level in the patient data management system and the calibration entry of more than 9 mg/dL, the correct blood glucose level was used in the accuracy analysis. The subsequent pairs, though, were excluded from the accuracy analysis since these were influenced by the preceding entry.

For reporting point accuracy, we used analytical and clinical accuracy measures: that is, Bland-Altman plot with bias and limits of agreement (bias ± 1.96 × standard deviation of the bias) [30], glucose prediction errors, and Clarke error grid analyses [31]. According to International Organization for Standardization (ISO) criteria, 95% of the paired measurements should be within the glucose prediction error criteria; the consensus is that 95% of the values should be in zones A and 5% in zones B of Clarke error grid analyses. Finally, we expressed the linearity between the device glucose results and blood glucose results by the Pearson correlation coefficient and coefficient of determination, R^2^.

In a *post hoc* analysis, we also report point accuracy according to the recently published consensus recommendations [1]. In this round-table meeting of ICU experts in blood glucose control, it was recommended to always report the mean absolute relative difference (MARD) when testing a CGM device, where MARD values should be less than 14%; values of more than 18% should be considered to represent poor accuracy [32]. We added the MARD as a *post hoc* analysis. Furthermore, we analyzed the point accuracy following the recently published surveillance error grid [33]. For more details, see Additional file 1.

We also reported reasons for early disconnection, defined as the removal of a sensor before 72 hours. For details, see Additional file 1. The time between calibrations using an incorrect glucose value entry and the next calibration was extracted from the total connection time of the device. Definitions of the metrics used to assess device reliability, including those suggested by recent consensus recommendations [1], are described in Additional file 1.

### Statistical analysis

We reported data as mean (± standard deviation) or median (interquartile range, or IQR) where appropriate. To be considered for the statistical analysis, each patient needed to have at least four comparative blood glucose results for accuracy analysis. However, the excluded patients remained included in the reliability analysis.

In a *post hoc* analysis, we used a linear mixed model to determine which variables influence the accuracy of the device. In addition, we stratified the accuracy results by diabetic status. For a detailed description of this model, see Additional file 1. Analyses were performed by using R (version: 2.15.1; R Foundation for Statistical Computing, Vienna, Austria).

## Results

### Patients and sensors

**Figure 1:**
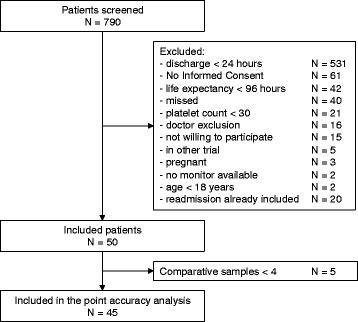
**CONSORT (Consolidated Standards of Reporting Trials) diagram of the study.**

We included 50 patients. In total, we used 105 sensors (median of 1 (IQR 1 to 3) sensor per patient) with a total connection time of 4,639 hours (median of 46 (IQR 27 to 134) hours per patient and median of 46 (IQR 22 to 69) hours per sensor). Five patients from whom we could not collect the minimum number of four consecutive comparative blood draws were excluded from the point accuracy analysis. A CONSORT (Consolidated Standards of Reporting Trials) diagram is provided in Figure 1. Patient characteristics and metrics of glucose control are shown in Tables 1 and 2.

**Table 1:** **Patient characteristics**

	**N = 50**	**N = 45**
	**All included patients**	**Patients included in the point accuracy analysis**
Age in years, median (IQR)	65 (56-72)	65 (55-72)
Male gender, number (%)	25 (50%)	24 (53%)
Race, number (%)		
Caucasian	45 (90%)	40 (89%)
Black	4 (8%)	4 (9%)
Asian	1 (2%)	1 (2%)
BMI in kg/m^2^, median (IQR)	24.7 (22.4-27.6)	24.4 (22.2-27.3)
Admission diagnosis, number (%)		
Medical	31 (62%)	26 (58%)
Emergency surgery	11 (22%)	11 (24%)
Planned surgery	8 (16%)	8 (18%)
Planned admission, number (%)	10 (20%)	9 (20%)
History of diabetes, number (%)		
No diabetes	39 (78%)	34 (76%)
Diabetes, unknown treatment	2 (4%)	2 (4%)
Diabetes treated with insulin	4 (8%)	4 (9%)
Diabetes treated with oral agents	5 (10%)	5 (12%)
APACHE II score, median (IQR)	23 (17-26)	22 (17-25)
SAPS II, median (IQR)	46 (39-55)	46 (40-55)
ICU LOS in days, median (IQR)	9 (4-16)	11 (5-16)
Hospital LOS in days, median (IQR)	19 (10-35)	19 (11-35)
ICU mortality, number (%)	11 (22%)	10 (22%)
Hospital mortality, number (%)	15 (30%)	14 (31%)

APACHE II, Acute Physiology and Chronic Health Evaluation II; BMI, body mass index; ICU, intensive care unit; IQR, interquartile range; LOS, length of stay; SAPS II, Simplified Acute Physiology Score II.

**Table 2:** **Measures of blood glucose control in patients included in point accuracy analysis**

Number of measurements	929
Mean blood glucose level per patient in mg/dL, median (IQR)	132 (125-148)
Standard deviation of blood glucose level per patient in mg/dL, median (IQR)	24 (16-33)
Number of measurements per patient, median, (IQR)	11 (7-29)
Severe hypoglycemia ≤40 mg/dL in measurements, number (%)	3 (0.3%)
Severe hypoglycemia ≤40 mg/dL in patients, number (%)	2 (4.4%)
Mild hypoglycemia 41-70 mg/dL in measurements, number (%)	15 (1.6%)
Mild hypoglycemia 41-70 mg/dL in patients, number (%)	7 (15.6%)
Mild hyperglycemia 150 -179 mg/dL in measurements, number (%)	163 (17.5%)
Mild hyperglycemia 150-179 mg/dL in patients, number (%)	35 (77.8%)
Severe hyperglycemia >180 mg/dL in measurements, number (%)	111 (11.9%)
Severe hyperglycemia >180 mg/dL in patients, number (%)	19 (42.2%)

Data consider all paired measurements, and result of blood gas analyzer is shown. IQR, interquartile range.

We could not inspect the insertion site of three sensors. Major bruises were observed in 3 out of 102 inspected insertion sensor sites; 10 minor bruises were seen. Major redness of the skin was observed in 7 out of 102 insertion sensor sites, and minor redness in 6 out of 102 insertion sites. Swelling of the skin was never seen, and none of the conscious patients mentioned pain at the sensor insertion site.

### Point accuracy

**Figure 2:**
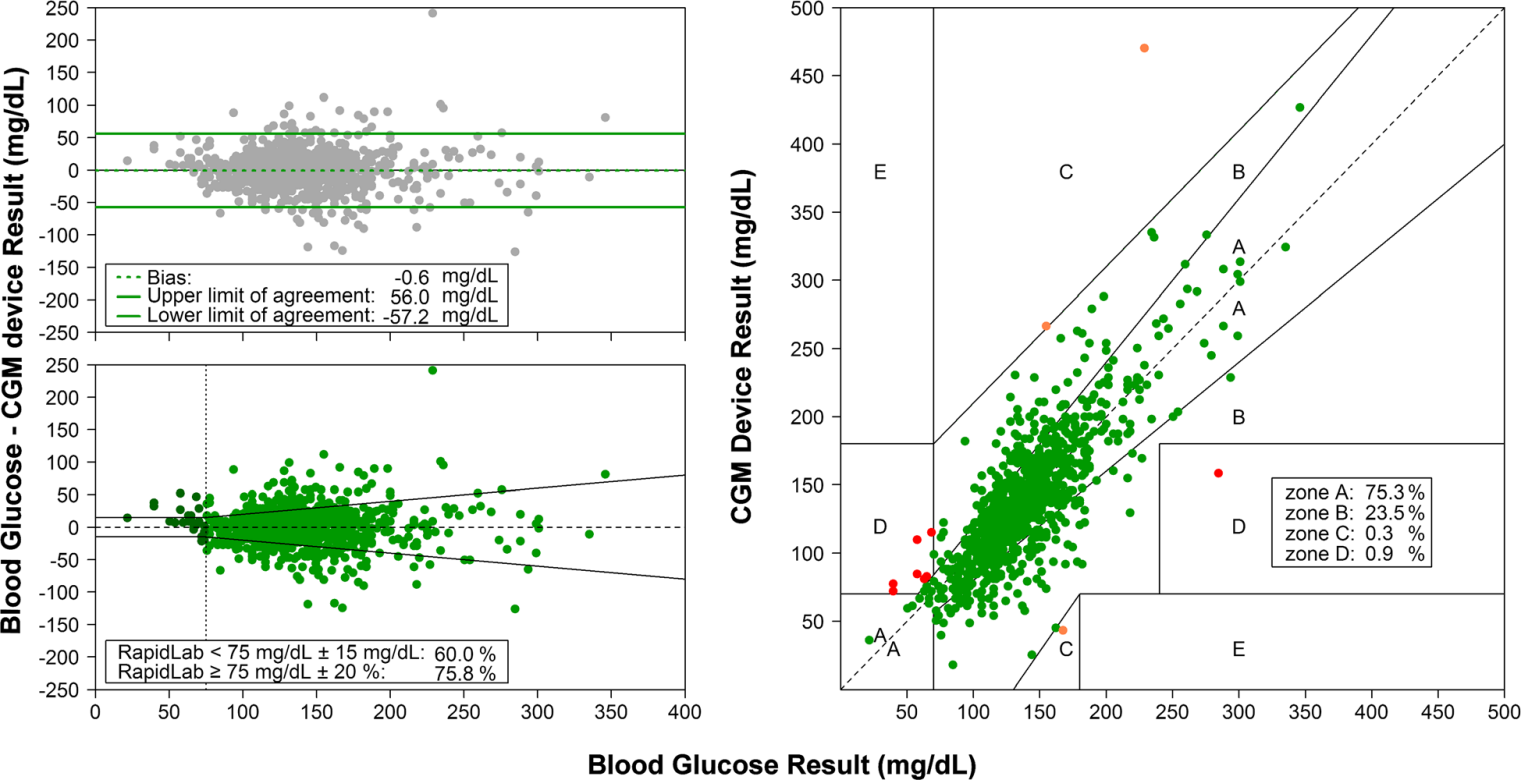
**Bland-Altman plot with bias and limits of agreement (bias ± 1.96 × standard deviation of the bias), glucose prediction errors, and Clarke error grid analyses.**

**Figure 3:**
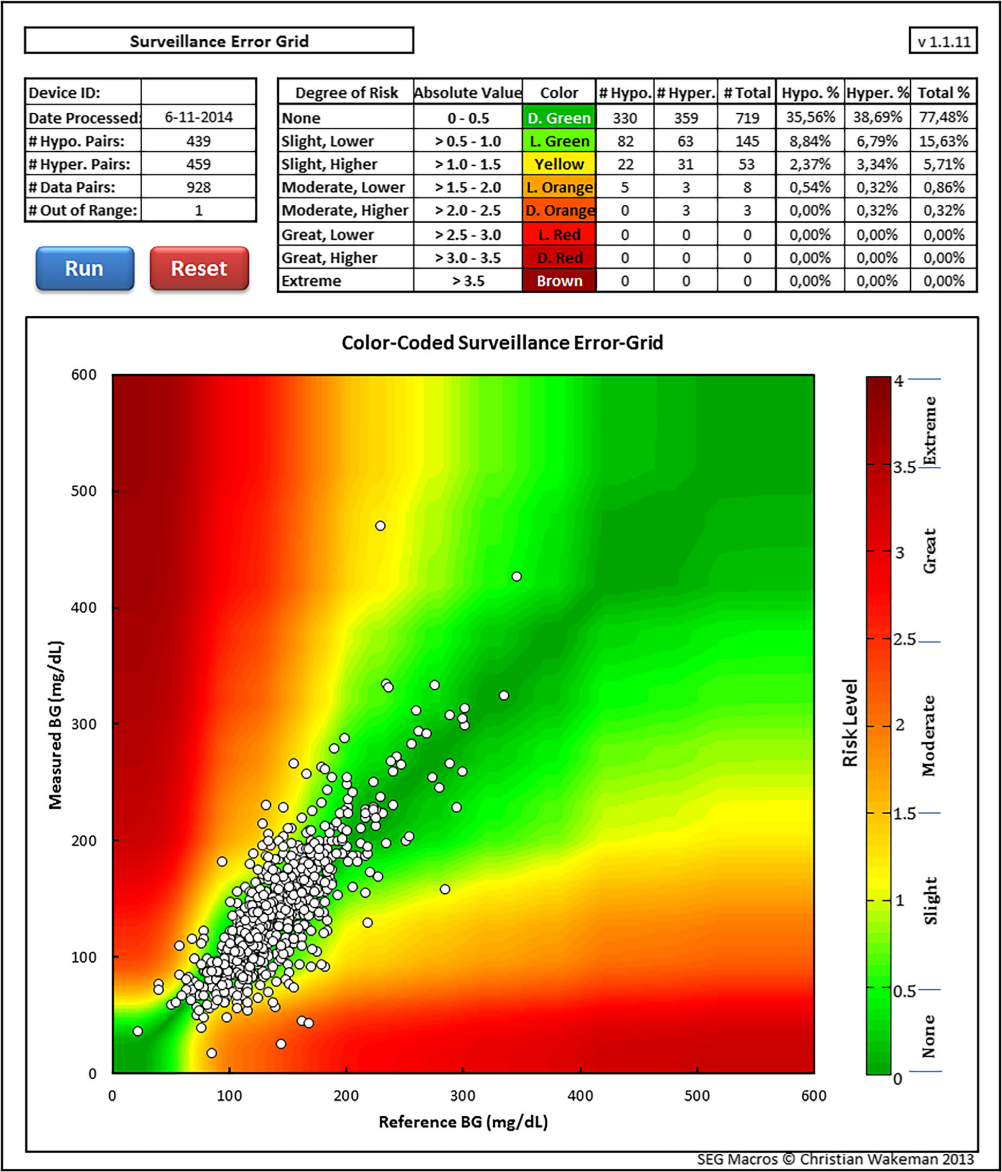
**Surveillance error grid with risk scores.**

We collected 929 comparative samples (11 (IQR 7 to 28) samples per patient). Bland-Altman plot, glucose prediction error grid, and Clarke error grid are presented in Figure 2. The surveillance error grid is presented in Figure 3. The Pearson correlation coefficient was 0.81; the R^2^ was 0.65. The MARD was 14.8%.

Fifty-eight percent of the device results were within 12.5% of the arterial blood glucose results (or within 10 mg/dL for results of less than 99 mg/dL), and 75% were within 20% of the arterial blood glucose results.

In the linear mixed model, only history of diabetes (*P* = 0.02) and number of calibrations per sensor (*P* = 0.04) affected the absolute difference between blood glucose and device result. Per each new calibration, the absolute difference decreased by 1.4% (standard error of 0.006%), meaning that the sensor performance increased. The effect of a history of diabetes was larger since an increase by 34.3% (standard error of 13.0%) in the absolute difference was found when comparing patients with a history of diabetes and patients without diabetes. In addition, we stratified the accuracy results by diabetic status; results are shown in Additional file 1: Figure S1 and Table S2. For detailed results of the multivariate random intercept model, see Additional file 1.

### Reliability of the continuous glucose-monitoring device

Start-up time after placing a new sensor was 49 (IQR 43 to 58) minutes. The number of skips in data acquisition was low, resulting in availability of real-time data during 95% (IQR 89% to 98%) of the connection time per sensor. Table 3 summarizes reliability metrics of the investigational device.

**Table 3:** **Device reliability**

	**Per patient**	**Per sensor**
Total number of sensors used	-	105
Number of sensors used, median (IQR)	1 (1-3)	-
Total connection time in hours, median (IQR)	46.2 (26.8-134.2)	45.8 (21.1-69.1)
Start-up time in minutes, median (IQR)	Median 49 (44-58)	49 (43-57.9)
Initialization time in minutes, median (IQR)	Median 34 (34-34.5)	34 (34,35)
Real-time data in hours	42.3 (23.1-130.3)	41.4 (20.6-64.0)
Percentage of real-time data, median (IQR)	94.1 (88.9-97.1)	94.6 (88.7-97.9)
Time of skips in data acquisition in hours, median (IQR)	4.3 (1.2-9.1)	2.6 (0.6-5.4)
Time of skips in data acquisition in hours caused by poor sensor signal, median (IQR)	0 (0-1.0)	0 (0-0.2)
Time of skips in data acquisition in minutes caused by other reasons, median (IQR)	3.3 (0.9-8.4)	2.0 (0.4-3.7)
Percentage of time of skips in data acquisition, median (IQR)	5.9 (2.9-11.1)	5.4 (2.1-11.3)
Percentage of time of skips in data acquisition in caused by poor sensor signal, median (IQR)	0 (0-0.7)	0 (0-0.3)
Percentage of time of skips in data acquisition caused by other reasons, median (IQR)	4.2 (2.3-8.0)	3.8 (1.5-8.0)
Number of calibrations, median (IQR)	14 (9-34)	12 (7-16)
Number of mandated calibrations, median (IQR)	8 (4-20)	6 (4-8)

IQR, interquartile range.

Out of 105 sensors, 60 were removed before 72 hours; the reasons for removal and the connection times of sensors are shown in Table 4. Out of 105 sensors, 42 were removed before 72 hours after insertion for reasons other than ICU discharge or death, and 36 sensors were removed because of an unresolved ‘Poor Sensor Signal’ alert or a device error (19 with no attempt to resolve).

**Table 4:** **Sensors removed less than 72 hours**

Total number of sensors used	105		
Total number of sensors removed <72 hours	60		
	**Number of sensors**	**Percentage of sensors removed <72 hours**	**Percentage of total number of sensors used**
Patient-related factors	18	(30%)	(17%)
Discharge <72 hours after insertion	14	(23%)	(13%)
Death 72 hours after insertion	4	(7%)	(4%)
Sensor- or device-related factors	42	(70%)	(40%)
Accidental removal of sensor	6	(10%)	(6%)
Poor sensor signal (19 had no attempt to resolve)	34	(57%)	(32%)
Device error	2	(3%)	(2%)
Duration of sensors in place in hours, median (IQR)			
All sensors	46 (21-69)		
Sensor that were removed <72 hours	22.1 (13.7-35.3)		
Sensors that were removed <72 hours because of patient-related factors	27.0 (21.3-41.47)		
Discharge <72 hours after insertion	24.7 (21.0-42.6)		
Death 72 hours after insertion	30.4 (26.5-34.8)		
Sensors that were removed <72 hours because of patient-related factors	18.9 (9.9-31.7)		
Accidental removal of sensor	29.4 (20.7-30.7)		
Poor sensor signal	19.4 (11.3-32.8)		
Device error	8.0 (7.4-8.5)		

IQR, interquartile range.

## Discussion

We determined the point accuracy and reliability of a device specifically designed for continuous real-time monitoring of interstitial glucose levels in critically ill patients. The analytic point accuracy of the device was low in a typical cohort of patients from a mixed medical-surgical ICU, according to ISO criteria and consensus recommendations. The clinical point accuracy was low according to Clarke error grid analysis but better according to surveillance error grid analysis. The device had few downtimes, but one third of the sensors were removed prematurely because of sensor- or device-related problems.

The present findings are in line with results from a previous trial testing the same device in cardiac surgery patients [34]. In that study, the mean absolute relative difference was 12.2% with 95% real-time data. Similar results come from studies testing other devices for interstitial glucose monitoring that were originally designed for use in non-critically ill patients. Those studies were performed in cardiac surgery patients [21,24,35], surgery patients [26], patients with neurologic emergencies [27], and non-surgical patients [16,22,25], and only two reported more favorable accuracy results [21,22]. Taken together, these data suggest that point accuracy of interstitial glucose monitoring cannot replace blood glucose level measurements.

In contrast to our findings, a previous publication by Brunner *et al.* [18] suggests a better point accuracy of another interstitial CGM device in critically ill patients. This report combined data of two separate trials in medical ICU patients [19,36]. The tested device in that study was from the same manufacturer but was not specifically designed for use in critically ill patients. In addition, the sensor was used for up to 72 hours and never replaced. One important difference with the present study was that the sensors were placed exclusively under the skin of the abdomen in patients included in these two trials. In most other trials, sensors were inserted under the skin of the abdomen [16,18,22,24,26,28], thigh [25,26], or shoulder [21]. Reported point accuracies do not suggest superiority of one of these sites. Certainly, there could be other unknown and unreported factors that could have resulted in the differences in performance.

We performed a mixed linear model to determine which factors could have influenced the point accuracy of the tested sensor. Rank order of measurement and presence of a history of diabetes affected the accuracy. The finding that rank order of measurement improved sensor performance is not new [18] and certainly is not surprising: more calibrations may always increase accuracy of a sensor. A history of diabetes was the most important variable influencing point accuracy and deteriorated sensor performance by 34%. As yet, this effect remains unexplained. It could be that microcirculation alteration in patients with diabetes affects interstitial glucose level. However, in previous studies with interstitial devices, diabetes was not found to be significantly associated with poor sensor accuracy in critically ill and cardiac surgery patients [16,18,28]. Moreover, in a recent study in cardiac surgery patients, an impaired microcirculation did not affect accuracy of two interstitial glucose sensors from two different manufacturers [28]. The difference found between patients with and without diabetes might also be related to glucose variability. Patients with diabetes will have more glucose variability compared with patients without diabetes. Thereby, when the focus is percentage difference, a greater disparity could be found when variability differences are compared.

It should be stressed that we compared interstitial glucose measurements with glucose levels in arterial blood samples, which are far from comparable. Indeed, the interstitial glucose level is dependent on several factors other than the blood glucose level, such as the speed of glucose diffusion from blood to interstitial spaces, as well as the rate of glucose uptake by subcutaneous cells [37]. Importantly, these factors are not constant, particularly in critically ill patients. Furthermore, there is a time lag between interstitial glucose and blood glucose measurement [37]. Studies suggested that the interstitial glucose level decreases before the blood glucose decreases [37,38], although this was not confirmed in other studies [39]. It is probably very difficult, if not impossible, to correct for factors causing a difference between interstitial and arterial blood glucose levels. Moreover, it is unknown whether differences between arterial and interstitial glucose levels are physiological.

Nevertheless, subcutaneous glucose monitoring could have advantages. One potential advantage is that continuous monitoring of interstitial glucose levels enables detection of trends in the blood glucose level [32]. This could allow earlier responses to a rise or a decline of the blood glucose level. In both cases, knowledge of the direction of the trend may be more valuable than the exact blood glucose level.

It is clear that the tested device can never replace blood glucose measurements. First, initial calibrations are always necessary, as are calibrations every 8 hours thereafter. As nurses were allowed to perform additional blood glucose measurements and as we asked them to insert the values into the investigational device monitor where they were used for additional calibrations, the number of calibrations in this study was higher than mandated. In fact, this could have improved the accuracy of the investigational device: it is possible that with fewer calibrations, point accuracy becomes worse.

Our trial has several strengths and weaknesses. Strengths include the fact that we were able to use the sensors for several days in the participating patients. Moreover, we used accurate blood gas analyzer measurements for comparisons as well as for the calibrations. Furthermore, we were able to test the device in a typical mixed medical-surgical ICU. Weaknesses include the small sample size and the single-center design of the trial. Furthermore, we did not collect as many samples as we expected. A more important limitation of our trial, though, is that the vast majority of blood glucose levels were in a narrow range, preventing us from drawing firm conclusions regarding accuracy in the hypoglycemic range. Although the ICU nurses were not allowed to change insulin infusion rates, they could have anticipated hypoglycemia by performing new blood glucose measurements earlier than dictated by the local guideline for blood glucose control, allowing them to respond earlier to, for example, hypoglycemia. Still, some hypoglycemic events occurred, probably because not all nurses were paying attention to the readings of the investigational device. In addition, nurses could have noted that its point accuracy was not always good, so they could have mistrusted the device readings. Finally, we cannot exclude the possibility that hypoglycemia can occur even with the use of CGM. The latter possibility will be the subject of a planned trial. An accuracy analysis limitation was that the assessment focused on percentage difference comparisons between the continuous sensor and discrete reference points, evaluated by standards meant for discrete measurements for dosing. Another important limitation is that trend accuracy was not evaluated. Trending is the most interesting endpoint but mandates very short intervals (that is, a short as 15 minutes) between blood glucose reference measurements [32,40]. Trend accuracy should and will be evaluated in future studies.

Notably, length of stay in the ICU and sensor connection time were far from similar. This was caused by the fact that sensors could not be used before informed consent was obtained. Thus, we may have missed an important phase of glucose control (that is, the first day or days of stay in the ICU). In addition, because of sensor- or device-related factors, one third of the sensors were removed before sensor life ended. This is an important problem for the reliability of the device. However, nurses did not always attempt to solve sensor- or device-related problems that could have been solved. During conduct of the trial, they were always allowed to remove the sensor because of ‘Poor Sensor Signal’ alerts or recurrent alarms. With increasing device-specific experience, it could be that there are fewer early removals.

## Conclusions

The point accuracy of a device designed for continuous real-time monitoring of the interstitial glucose level did not meet the ISO15197 standard or the recent consensus guidance for discrete glucose measurement for dosing when used on critically on critically ill patients admitted to a mixed medical-surgical ICU. Although this device is not a replacement for current blood gas analyzer measurements, a real-time system may be used for trend guidance on timely reference measurement for insulin adjustment. The device had few downtimes, but one third of the sensors were removed prematurely because of unresolved sensor- or device-related problems.

## Key messages

- An interstitial glucose sensor system in critically ill patients cannot replace blood glucose level measurements but may provide important trend information for glucose management.
- Sensors are frequently removed prematurely for a variety of reasons.

## Additional file

Additional file 1:
**Extended method section describing the local guideline for glucose control, methods to calculate point accuracy, definitions of metrics for device reliability.** *Post hoc* analysis about factors that affect point accuracy including **Table S1** with results from the linear mixed model, **Table S2** with accuracy metrics stratified by diabetic status, and **Figure S1** with Bland-Altman plot with bias and limits of agreement (bias ± 1.96 standard deviation of the bias), glucose prediction errors, and Clarke error grid analyses stratified by diabetic status.

## Abbreviations

CGM: continuous glucose monitoring
ICU: intensive care unit
IQR: interquartile range
ISO: International Organization for Standardization
MARD: mean absolute relative difference
