# Supplementary material for: Point accuracy and reliability of an interstitial continuous glucose-monitoring device in critically ill patients: a prospective study
Source: Crit Care. 2015 Feb 5;19(1):34. doi: 10.1186/s13054-015-0757-4 (PMC4382831; doi:10.1186/s13054-015-0757-4)
Supplement: Additional file 1: — Extended method section describing the local guideline for glucose control, methods to calculate point accuracy, definitions of metrics for device reliability. Post hoc analysis about factors that affect point accuracy including Table S1 with results from the linear mixed model, Table S2 with accuracy metrics stratified by diabetic status, and Figure S1 with Bland-Altman plot with bias and limits of agreement (bias ± 1.96 standard deviation of the bias), glucose prediction errors, and Clarke error grid analyses stratified by diabetic status. [file 13054_2015_757_MOESM1_ESM.pdf]

Supplementary Appendix to "Point Accuracy and Reliability of an Interstitial Continuous Glucose Monitoring Device in Critically Ill Patients: a Prospective Study"

Roosmarijn T.M. van Hooijdonk[1], Jan Hendrik Leopold[1], Tineke Winters[1], Jan M. Binnekade[1], Nicole P. Juffermans[1,3], Janneke Horn[1,3], Johan C. Fischer[2], Edmée C. van Dongen–Lases[2], Marcus J. Schultz[1,3]

Academic Medical Center, University of Amsterdam, Amsterdam, the Netherlands: [1]Department of Intensive Care; [2]Department of Clinical Chemistry; [3]Laboratory of Experimental Intensive Care and Anesthesiology (L·E·I·C·A)

The local guideline for glucose control

ICU nurses performed glucose control with insulin, following a local guideline for blood glucose control targeting a blood glucose level between 90–144 mg/dL [29].

According to this local guideline, insulin infusion was started when the blood glucose level was > 144 mg/dL. Insulin titration adjustments were made based on sliding scales. The guideline advised to stop insulin infusion and to give boluses of dextrose only when the blood glucose level declined to < 61 mg/dL. Insulin infusion was exclusively given intravenously and continuously; boluses of insulin were only allowed when the blood glucose level was > 360 mg/dL; subcutaneous insulin boluses were never allowed.

The local guideline also dictated to perform blood glucose measurements at least every four hours, but more frequently if blood glucose levels were out of range or rapidly changing. Typically, blood glucose levels were measured more frequently at the start of insulin titration, and in cases of an increased risk of hypoglycemia. Blood glucose levels used for insulin adjustment were measured in arterial blood samples using RapidLab 1265 blood gas analyzers (Siemens Healthcare Diagnostics, The Hague, The Netherlands), located in the ICU. The results were automatically downloaded to the patient data management system (MetaVision®, iMDsoft, Tel Aviv, Israel), which was present at every ICU bed.

Methods to calculate point accuracy

For reporting point accuracy we used glucose prediction errors, defined as [blood glucose – device glucose result]. The percentage of data points that fell within ± 15 mg/dL of the blood glucose results for blood glucose results < 75 mg/dL and within 20% of the blood glucose results for blood glucose results ≥ 75 mg/dL were reported

according to the current International Standards Organization standard (ISO15197) [32]. We also used Clarke error grid analyses to show the percentage of paired data values falling within each zone of the Clarke error grid [30], and Bland–Altman plot [31]. The Clarke error grid is divided in 5 paired ‘zones’: zones A (measurement within 20% of the reference or glucose levels < 70 mg/dL); zones B (measurement more than 20% different from the reference but still clinically acceptable as they would not change the rate of insulin infusion); zones C (measurement that would lead to unnecessary changes in insulin infusion, i.e., overcorrecting acceptable glucose levels); zones D (potentially dangerous hypo– or hyperglycemic events are missed); and zones E (levels that would lead to a decision opposite to that required, i.e., treatment for hypoglycemia instead of hyperglycemia). General consensus is that 95% of the values should be in zones A and 5% in zones B.

The Bland–Altman plot is presented with bias (mean difference between the device glucose results and blood glucose results) and limits of agreement (bias ± 1.96 x standard deviation of the bias) to analyze the agreement between the device glucose results and blood glucose results.

In a post–hoc analysis we also determined point accuracy according to the recently published consensus recommendations [1]. For this, the percentage of data points that fell within 12.5% of the blood glucose results, or within 10 mg/dL for readings < 99 mg/dL were reported. In a round the table meeting of ICU experts it was recommended to report the mean absolute relative difference and values should be <14%; values >18% were considered to represent poor accuracy [32]. Furthermore, we analyzed the accuracy following the recently published the surveillance error grid [33].

Definitions of metrics for device reliability

The following metrics and definitions were used to assess device reliability, including those suggested by recent consensus recommendations [1]

- Connection time – time between first device glucose results and last glucose result
- Start–up time – time between the start of initialization of sensor and first device glucose result after calibration, including blood glucose measurement time and time for nurse to enter value into the device.
- Initialization time – time between initialization of sensor and ready for calibration
- Real-time data – time when device glucose results were available
- Percentage of real-time data – percentage of time device glucose results were available divided by total connection time
- Skips in data acquisition all causes – total time when the monitor gave no results
- Percentage of skips in data acquisition all causes – percentage of time when the monitor gave no results divided by the connection time
- Skips in data acquisition poor sensor signal – percentage of time when the monitor gave no results caused by poor sensor signal
- Percentage of skips in data acquisition poor sensor signal – percentage of time of skips in data acquisition caused by poor sensor signal device divided by the connection time minus the time of skips in data acquisition caused by other reasons
- Skips in data acquisition other reasons – time of skips in data acquisition caused by other reasons than poor sensor signal

- Percentage of skips in data acquisition other reasons – percentage of time of skips in data acquisition caused by other reasons divided by the connection time minus time of skips in data acquisition caused by poor sensor signal
- ‘Poor Sensor Signal’ – a device alert indicating that the sensor may be experiencing decreased performance. This alert removes the real time sensor glucose value display until a requested reference calibration value is entered to recover sensor performance.

Factors that Affect Point Accuracy

Background

The aim of the primary study was to the test the point accuracy and reliability of an interstitial CGM device in a mixed medical–surgical ICU. We found a low point accuracy of an interstitial CGM device in a mixed medical–surgical ICU. We were interested if this was dependent on particular variables. Therefore, we performed a post-hoc analysis to determine which variables influence the accuracy of the device.

Methods

We used a linear mixed model to determine which variables influence the accuracy of the device. For this, patient and sensor were used as random intercepts to account for repeated measurements. The absolute difference between the arterial blood glucose level and device glucose level was the dependent variable. The absolute difference was logarithmically transformed (using the natural logarithm) to obtain a normal distribution. The following variables were chosen based on clinical relevance and previous trials testing other CGM devices [18, 19, 28]: demographic variables including gender, age, body mass index and history of diabetes; disease severity variables including the APACHE II score and the circulation score of the Sequential

Organ Failure Assessment (SOFA) Score on the day of measurements; in addition, we added time between calibrations (as shorter time between calibrations could improve accuracy) and the rank order of the paired glucose results (as more calibrations could improve accuracy) [18]. All variables were added to the model without considering further model reduction strategies. Visual inspection of residuals was done. Correlation between covariates was assessed to investigate collinearity. The effect of covariates on the absolute difference was reported as the percentage of change in the absolute difference with the standard error.

Results (table S1)

We performed a linear mixed-effects model with a fit by maximum likelihood. Visual inspection of residual plots did not reveal any obvious deviations from homoscedasticity or normality. Pearson correlation coefficients were all under 0.5 showing no collinearity.

In the linear mixed model only history of diabetes (P = 0.02) and number of calibrations per sensor (P = 0.04) affected the absolute difference between blood glucose and device result. Per each new calibration the absolute difference decreased with 1.4% (standard error of 0.006%), meaning that the sensor performance increased. The effect of a history of diabetes was bigger, though, since diabetes increased the absolute difference with 34.3% (standard error of 13.0%). Therefore we stratified our accuracy metrics by diabetic status (see figure S1 and table S2).

The formula for the final mixed model was:

Log(Absolute difference) = 2.419+ random intercept per patient + random intercept per sensor + 0.295*Diabetes -0.014*rank order of measurement+ 0.011* Sofa

Circulation Score + 0.025*BMI -0.073*Gender -0.003* Age -0.009*APACHE II + 0.0001*time between calibration

Table S1

Random effects	Variance	Standard deviation	
Patient ID	0.036	0.189	
Sensor ID	0.047	0.217	
**Fixed effects**	**Value**	**Standard error**	**P-value**
Intercept	2.419	0.489	0.000
History of Diabetes	0.295	2.408	0.021
Rank order	-0.014	0.007	0.037
Sofa circulation score	0.011	0.026	0.681
BMI	0.025	0.015	0.107
Male gender	-0.073	0.109	0.506
Age in years	-0.003	0.004	0.524
APACHE II score	-0.009	0.007	0.204
Time between calibration in minutes	0.0001	0.0001	0.629

Table S2 Accuracy metrics stratified by diabetic status

	Diabetic	Non-diabetic	All patients
Number of paired samples	337	592	929
Mean absolute relative difference	16.0	14.2	14.8
Correlation coefficient	0.84	0.71	0.81
R^2	0.70	0.50	0.65
Consensus recommendations			
- percentage of measurements within 12.5% blood glucose results (or within 10 mg/dL for results < 99 mg/dL)	55	59	58
- percentage of measurements within 20% blood glucose results (	72	77	75

Figure S1: Bland–Altman plot with bias and limits of agreement (bias ± 1.96 standard deviation of the bias), glucose prediction errors, and Clarke error grid analyses stratified by diabetic status.

Patients without an history of diabetes

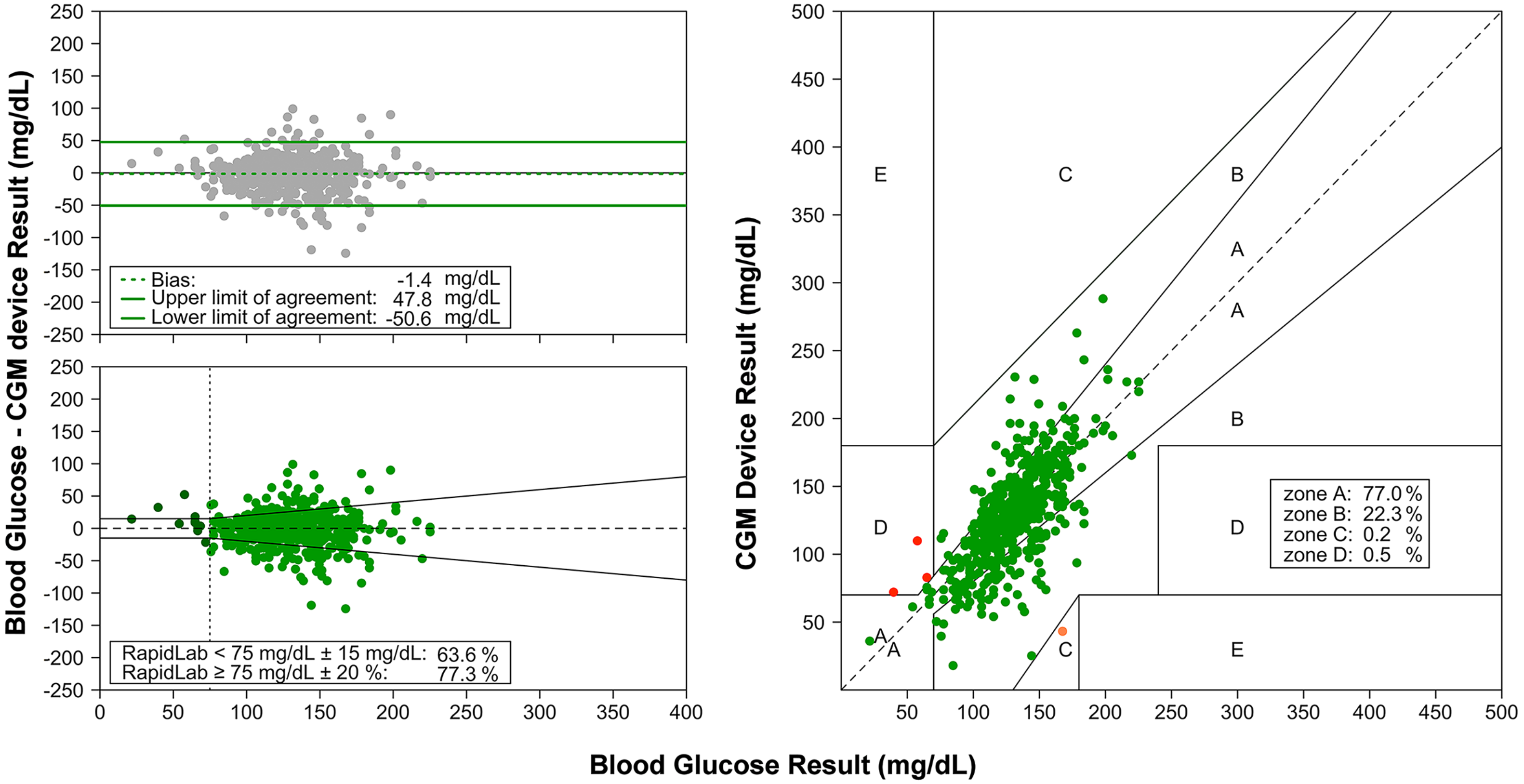

Patients with an history of diabetes

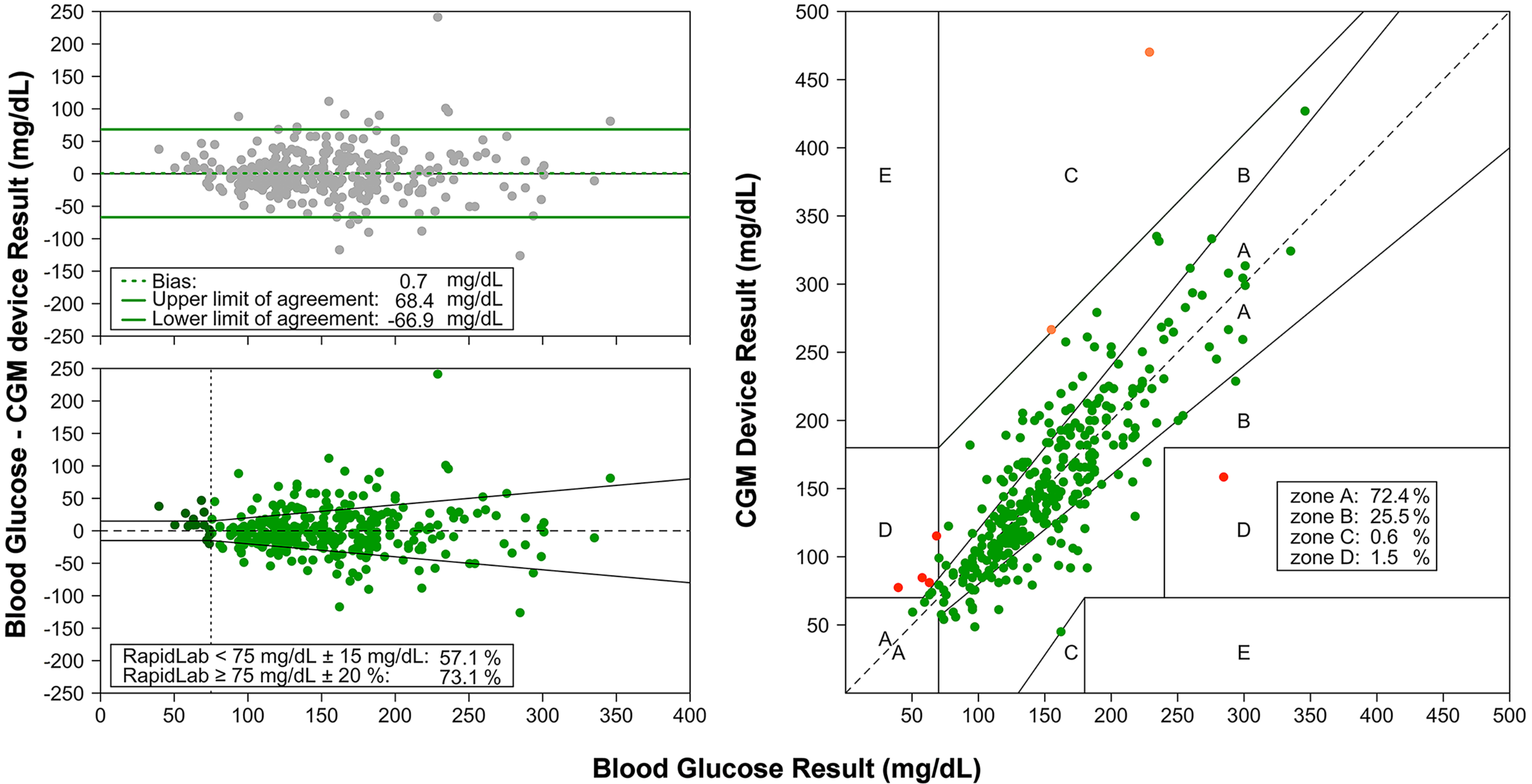

Reliability Analysis

Background

In the present study we found that more than half of the sensors had to be removed before 72 hours. We wanted to know reasons for disconnection and when this happened. Therefore we did a post–hoc analysis to investigate reasons for early disconnection.

Methods

Early disconnection was defined as the removal of a sensor before 72 hours, which could be caused by:

- Poor sensor signal – sensor performance issue, in which the systems requests additional calibrations to solve. Nurses were able to remove the sensor when the monitor gave a poor sensor signal alarm without attempt to solve.
- Accidental removal of the sensor
- Device error – the device monitor had an technical failure

Furthermore the connection time was calculated (time between first device glucose results and last glucose result) for sensors, which were removed before 72 hours. The time between calibrations using an incorrect glucose value entry and the next calibration was extracted from the total connection time of the device.
